# Microbiome in Healthy Women Between Two Districts With Different Air Quality Index

**DOI:** 10.3389/fmicb.2020.548618

**Published:** 2020-10-19

**Authors:** Yinhua Wu, Zujin Wang, Yu Zhang, Liming Ruan, Ang Li, Xiaoyan Liu

**Affiliations:** ^1^ Department of Dermatology, The First Affiliated Hospital, School of Medicine of Zhejiang University, Hangzhou, China; ^2^ Department of the Second General Surgeon, The Yunhe People’s Hospital, Yunhe, China; ^3^ Physician Health Center, The First Affiliated Hospital of Zhengzhou University, Zhengzhou, China; ^4^ Department of Henan Gene Hospital, The First Affiliated Hospital of Zhengzhou University, Zhengzhou, China

**Keywords:** airborne pollution, air quality index, 16S rRNA gene, microbiome, healthy women

## Abstract

Although the diversity and abundance of skin microbiome are mainly determined by intrinsic factors, including gender, age, anatomical site, and ethnicity, we question whether facial microbiome could be affected by long-term exposure to airborne pollution. Using 16S ribosomal RNA (rRNA) gene amplicon sequencing, we analyzed the facial bacterial microbiome of healthy and young Chinese women (25–35 years old) between two districts with different air quality indices (AQIs) in Zhejiang Province. The overall microbiome structure was obviously different between these two districts. It revealed an increase in both the abundance and diversity of facial bacterial microbiome in Hangzhou (HZ) with higher AQI compared with those in Yunhe (YH) with lower AQI. Linear discriminant analysis (LDA) and Lefse analysis identified a total of 45 genera showing significant overrepresentation in the HZ group. Furthermore, PICRUSt analysis showed that functional pathways associated with metabolism of saturated fatty acid were relatively more predominant in the HZ group, whereas those with DNA repair or mitochondrial DNA replication were more predominant in the YH group. Our present data can provide useful information for further researches on the composition and function of the skin microbiome related to air pollution factors as well as for the development of therapeutic agents targeting the microbes and their metabolites to resist damages of airborne pollutants.

## Introduction

Human skin is the largest organ and acts as the first line of defense against environmental stressors. It harbors hundreds and thousands of resident microbes and maintains a delicate equilibrium, the microbiome, which is necessary for a healthy skin (Grice et al., 2009; Dréno et al., 2016). This balance is constantly influenced by both internal (host) and external (environmental) factors. These factors alter the composition of skin microbiome and further affect the barrier function of host skin.

With the rapid development of Chinese economy, environmental pollution and hazier weather have become a critical problem that cannot be overlooked. Air quality index (AQI) describes the extent of clean or polluted air and its impact on human health. The main air pollutants are mainly composed of particulate matter (PM_2.5_ or PM_10_), sulfur dioxide (SO_2_), nitrogen dioxide (NO_2_), ozone (O_3_), and carbon monoxide (CO; Araviiskaia et al., 2019), and they are also five major pollution standards to calculate the AQI (Woodall et al., 2017). It was reported that accumulation of SO_2_ and NO_x_ could result in a lower pH value of rainwater (Li et al., 2019), and difference of pH would further lead to composition change in local microbiota (Vongsa et al., 2019). Available evidence indicate that long-term air pollution could accelerate skin aging (Vierktter et al., 2010; Ding et al., 2017), and some human skin diseases, such as acne (Liu et al., 2018), atopic dermatitis (AD; Ahn, 2014), and eczema (Pesce et al., 2015), were also reported to be associated with dysbiosis of skin microbiota (Fitz-Gibbon et al., 2013; Chng et al., 2016; Dybboe et al., 2017; Shibagaki et al., 2017; Kim et al., 2019; Reiger et al., 2020). On the one hand, it was uncovered that exposure to atmospheric-equivalent O_3_ levels for 2 h could halve the skin microbiome of women’s forearm (He et al., 2006). On the other hand, skin microbiome is thought to remain stable over months to years once intrinsic factors stabilize (Oh et al., 2016), and the stability of healthy skin microbiome established in early period might extend into adulthood (Capone et al., 2011). There was a very little impact of short-term exposure to coal combustion waste on the skin microbiome of adult (Hughey et al., 2016). However, a study conducted in four cities with different urbanization in China reported that skin microbiome in cities with better environmental standards and higher socioeconomic status had higher overall and phylogenetic diversities, which would be beneficial to human skin health (Kim et al., 2018). Therefore, whether airborne pollutants, especially long-term exposure to them, can affect the equilibrium of skin microbiome of healthy individuals has not directly been evaluated and needs to be further investigated.

One host factor, body mass index (BMI), was reported to be closely related to oral or vulva microbiota (Vongsa et al., 2019; de Andrade et al., 2020) besides gut microbiota (Ottosson et al., 2018). In addition, age also would impact the facial microbiome (Kim et al., 2019). In order to reduce the influence of these host factors on skin microbiome and mainly focus on discussing the poorly understood relationships between the facial microbiome composition of healthy women and air quality, we recruited 25–35-year-old healthy women with a BMI of 18.5–24.9 from Hangzhou (HZ, Zhejiang Province, China) or Yunhe (YH, Zhejiang Province, China) districts with different AQIs. As another host factor, skin sites vary dramatically in their levels of bacterial composition (Costello et al., 2009), and site-specific lipid and water levels were the most significant predictors of facial microbiome composition and diversity (Mukherjee et al., 2016). Thus, we collected skin samples from facial oily zone (forehead, nasal dorsum, and jaw) or dry zone (cheek) and characterized their bacterial microbiome using 16S ribosomal RNA (rRNA) sequencing. We aimed to explore the effect and probable mechanism of atmospheric pollution on healthy human skin.

## Materials and Methods

### Participants and Sampling

This study was approved by the Institutional Review Board of the First Affiliated Hospital, School of Medicine of Zhejiang University (reference number 2017-866). Written informed consent was obtained from all participants. For comparison of skin bacterial microbiome characteristics between two districts with different AQIs, we recruited 20 healthy women aged 25–35 years old for each group from HZ or YH, respectively. All of the selected subjects have been living in HZ or YH for more than 20 years. They had no history of chronic cutaneous diseases, such as acne, psoriasis, AD, and eczema. In addition, subjects would be excluded if they answered “yes” to any of the exclusion criteria (Supplementary Table S1) according to core microbiome sampling protocol for Human Microbiome Project (McInnes and Cutting, 2010). All subjects only used the provided cleanser and lotions for 2 weeks prior to sampling in order to minimize the impact of cosmetics. All microbial samples were collected in the same week in May 2019, in temperature‐ and humidity-controlled rooms (22°C, humidity 60%), 2 h after the last face wash. The skin area used for sampling was the oily zone (forehead, nasal dorsum, and jaw) and the dry zone (3 × 3 cm^2^ for the cheek). The samples were collected with sterile Catch-All Sample Collections Swabs (QEC091H, Epicentre, USA) and stored at −80°C within 15 min after collection until genomic DNA (gDNA) extraction. We also collected the clinical parameters of the participants, including BMI and age. BMI was calculated by the equation of weight (kg)/height (m^2^) and categorized in the World Health Organization guidelines: normal weight, BMI of 18.5–24.9 kg/m^2^. In order to explore whether different AQIs could lead to change in facial pH value and further impact the facial microbiome, the skin surface pH was measured using a skin pH-meter_®_ pH905 (Courage + Khazaka Electronic GmbH, Germany).

Finally, 18 subjects from HZ and 11 subjects from YH were included due to discarding samples with low bacterial DNA. Among these 29 subjects, 10 subjects had both oily and dry-zone samples, so a total of 39 samples were used for further analysis. No significant differences in age were observed between these two groups, while the value of BMI in YH group tended to be a little higher (*p* = 0.035). The related clinical characteristics of tested subjects are shown in Table 1.

**Table 1:** Demographic and clinical details of recruited subjects.

Demographic and clinical indices		HZ	YH	*p*
		*n* = 18	*n* = 11	
Age (year, mean ± SD)		29.22 ± 3.85	28.46 ± 4.74	0.640
BMI (kg m^−2^, mean ± SD)		19.83 ± 1.20	21.35 ± 2.48	0.035^*^
Skin type		*n* %	*n* %	
	Combination	8 44.44	6 54.55	
	Oily	2 11.11	2 18.18	
	Dry	0 0.00	1 9.09	
	Neutral	8 44.44	2 18.18	
pH value (mean ± SD)	Oily zone	5.67 ± 0.19	5.75 ± 0.25	0.338
	Dry zone	5.92 ± 0.27	5.94 ± 0.87	0.928

* *n*, the number of included cases of each group; %, the percentage of each skin type. BMI, body mass index.

### Amplicon Generation, Sequencing, and Bioinformatics Analysis

DNA was extracted using PSP Spin DNA plus kit (Stratec, Berlin, Germany) according to manufacturer’s instructions. The V3–V4 region of 16S rRNA was PCR amplified with specific primers 341F 5'-CCTAYGGGRBGCASCAG-3' and 806R 5'-GGACTACNNGGGTATCTAAT-3' with the barcode. All PCR reactions were carried out in 30 μl reactions with 15 μl of Phusion® High-Fidelity PCR Master Mix (New England Biolabs), 0.2 μM of forward and reverse primers, and about 10 ng template DNA. Thermal cycling consisted of initial denaturation at 98°C for 1 min, followed by 30 cycles of denaturation at 98°C for 10 s, annealing at 50°C for 30 s, and elongation at 72°C for 30 s, and finally, 72°C for 5 min. PCR product was sequenced on Illumina Miseq sequencer. For bioinformatics analysis, refer to our previous study (Hu et al., 2019). Raw Illumina read data have been deposited in the EMBL database (study accession number PRJEB37433).

### Statistics Analysis

Statistical analyses were performed on R version 3.6.1. R program package “phyloseq” was used to caculate unifrac distances. R program package “vegan” was used to perform permutational multivariate analysis of variance (PERMANOVA) and to calculate Shannon, Simpson, Inv-simpon, and observed species. R program package “fossil” was used to calculate Chao1 and ICE index.

Wilcoxon rank sum test was used to perform hypothesis test of alpha diversity and principle coordinates. Lefse version 1.0 (Segata et al., 2011) was used to find out whether taxons and Kyoto Encyclopedia of Genes and Genomes (KEGG) modules might be significantly associated destinations with default parameters. The Lefse analysis results were deposited in https://github.com/xiaoshubaba/Liuxiaoyan_SKIN_16Smetagenome.

The results of age, BMI, values of airborne pollutants, and AQI were expressed as the mean ± standard deviation. T-tests using SPSS software were used to assess significant differences between the HZ and TH groups. *p* < 0.05 was considered to be statistically significant.

## Results

### Permutational Multivariate Analysis of Variance

PERMANOVA showed that the effect of destination (cohort) was strongly associated with microbial composition in all of five distances, including JSD, Pearson, Spearman, and weighted UniFrac and unweighted UniFrac distances. PERMANOVA (Supplementary Table S2) revealed a weak association between skin type and microbial composition only in one distance (unweighted UniFrac distance, *p* = 0.020), which was much less significant than that in the destination. Furthermore, there was no obvious association between BMI and microbial composition.

### Sequence Reads and Composition at the Whole Microbiome Level

A total of 1,041,112 qualified reads from 39 samples (mean, 26,695 ± 14,421) were sequenced, and 1,063 qualified operational taxonomy units (OTUs) were clustered for downstream analysis. For details, please refer to Supplementary Table S3.

Principal coordinate analysis (PCoA) was performed with five different distances at the OTU level. There was no significant difference on these principal coordinates between HZ and YH subjects (Supplementary Figure S1, *p* > 0.05).

### Facial Microbial Diversity Changes Between the HZ and YH Groups

**Figure 1:**
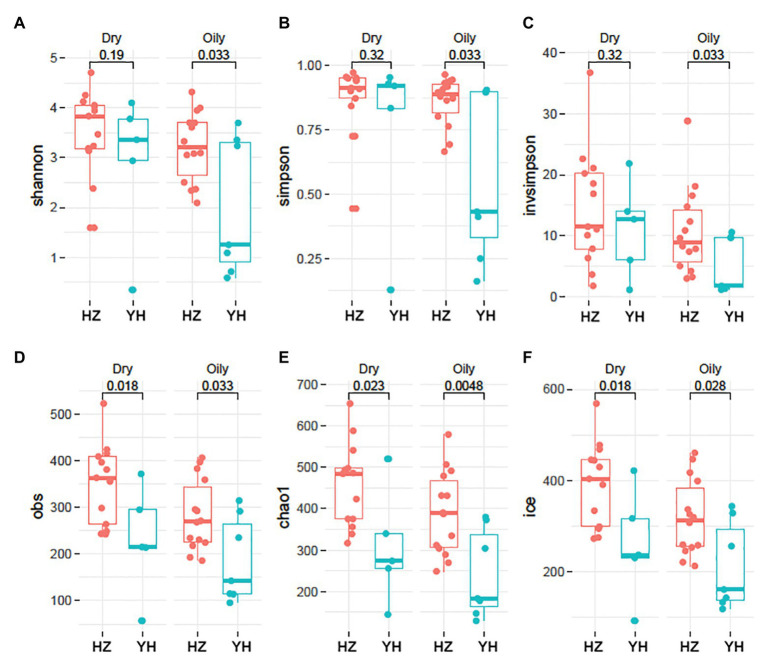
Phylogenetic diversity and richness of facial bacterial microbiome in HZ female compared to YH female individuals. **(A–F)** Alpha (*α*) diversity in Hangzhou (HZ) and Yunhe (YH) subjects. Oily represented samples from oily zone, and dry represented samples from dry zone. Each dot represented a sample. Shannon, Simpson, and inverse Simpson (invsimpson) indicate diversity indices, and Obs, Chao1, and Ice indicate microbial richness. Significance determined in Wilcoxon rank sum test with one-sided alternatives.

Shannon, Simpson, and inverse Simpson [invsimpson], which usually indicate diversity indexes, were used to assess facial microbial diversity. As shown in Figure 1, Shannon, Simpson, and Invsimpson index were greater in HZ oily zone compared with those in YH oily zone (*p* = 0.033, 0.033, and 0.033). However, no diversity changes in dry zone were observed between the two districts (*p* > 0.05). Furthermore, microbial richness in HZ subjects, as estimated by Obs, Chao1, and Ice, was higher than that in YH, not only in oily zone (*p* = 0.033, 0.048, and 0.028) but also in dry zone (*p* = 0.018, 0.023, and 0.018). It was revealed by these data that for the facial bacterial microbiome, the HZ group showed a higher alpha diversity and richness compared with the YH group.

Except for distribution, the value of *p* is also greatly impacted by sample size, so we used a bootstrap sampling (randomly chose five HZ dry-zone and seven HZ oily zone samples), then repeated this process for 100 times. We found that the majority of value distribution of *p* (Supplementary Figure S2) is still significant (median *p* < 0.05), although YH had only five dry‐ and seven oily zone samples.

### Differences in Taxonomic Composition of Facial Bacterial Microbiome Between the HZ and YH Groups

**Figure 2:**
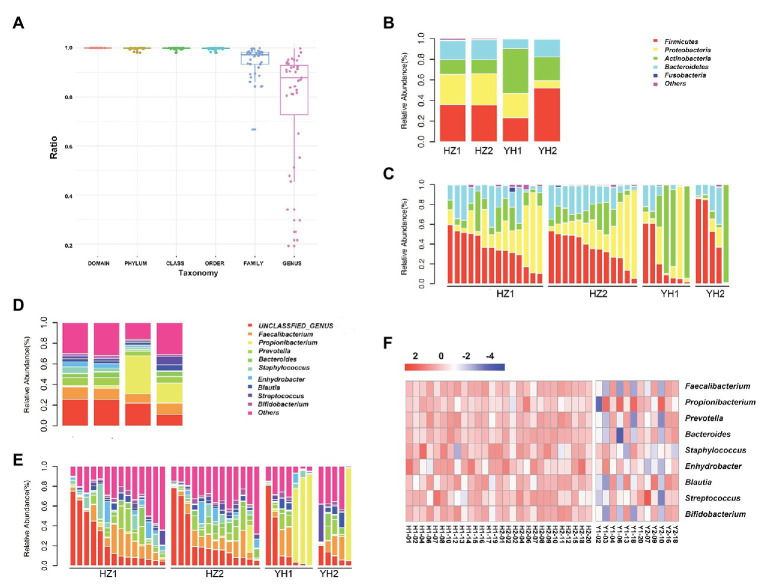
Taxonomy resolution of facial bacterial microbiome in healthy female individuals between the HZ and YH groups. **(A)** Taxonomy resolution in all samples; each bar represented ratio of reads annotated at that level. **(B,C)** Relative abundance levels of the phyla presented in samples from the HZ and YH healthy female individuals. **(D,E)** Relative abundance levels of genera presented in samples from the HZ and YH female individuals. **(F)** The heatmap of the most dominated genera of the facial microbiome. HZ1 and H1 indicated data of oily zone in the HZ district, HZ2 and H2 indicated data of dry zone in the HZ district, YH1 and Y1 indicated data of oily zone in the YH district, and YH2 and H2 indicated data of dry zone in the YH district.

We further investigated the taxonomic profiles in the destination-related diversification of facial microbiome. The taxonomy resolution of all samples is shown in Figure 2A, and the ratio at genus level was about 77.21%. As shown in Figures 2B,C, *Firmicutes*, *Proteobacteria*, *Actinobacteria*, and *Bacteroidetes* were the four phyla that composed the major facial bacterial microbiome. Further comparison of the phyla abundance in both groups uncovered that *Firmicutes* and *Proteobacteria* were the two most predominant in the HZ group, accounting for the average abundance of 35.63 and 30.37% in the oily zone and 35.97 and 29.27% in the dry zone, respectively. However, in the YH group, *Actinobacteria* was the most predominant (43.59%) in the oily zone, and *Firmicutes* accounted for 52.18% in the dry zone. Overall, these data indicated that the HZ group had a tendency to have more *Proteobacteria* and less *Actinobacteria* in the facial bacterial microbiome than the YH group.

In addition, *Faecalibacterium*, *Propionibacterium*, *Prevotellaceae*, *Bacteroides*, *Staphylococcus*, *Enhydrobacter*, *Blautia*, *Streptococcus*, and *Bigidobacterium* were found to be the top nine genera in all microbiome. Notably, a comparison at the genus level between the two groups showed that although value of *p* > 0.05, the abundance of *Propionibacterium* was much lower in the HZ group (1.78%) compared to the that in the YH group (29.43%), in both oily and dry zones (Figures 2D–F, Supplementary Table S4). It might partly interpret the marked reduction in *Actinobacteria* in the facial bacterial microbiomes of the HZ subjects since *Propionibacterium* is the major genus belonging to this phylum. In contrast, the abundance of *Bacteroides*, *Staphylococcus*, and *Enhydrobacter* were significantly increased in the HZ group compared to that in the YH group (*p* < 0.05, Figures 2D–F, Supplementary Table S4).

**Figure 3:**
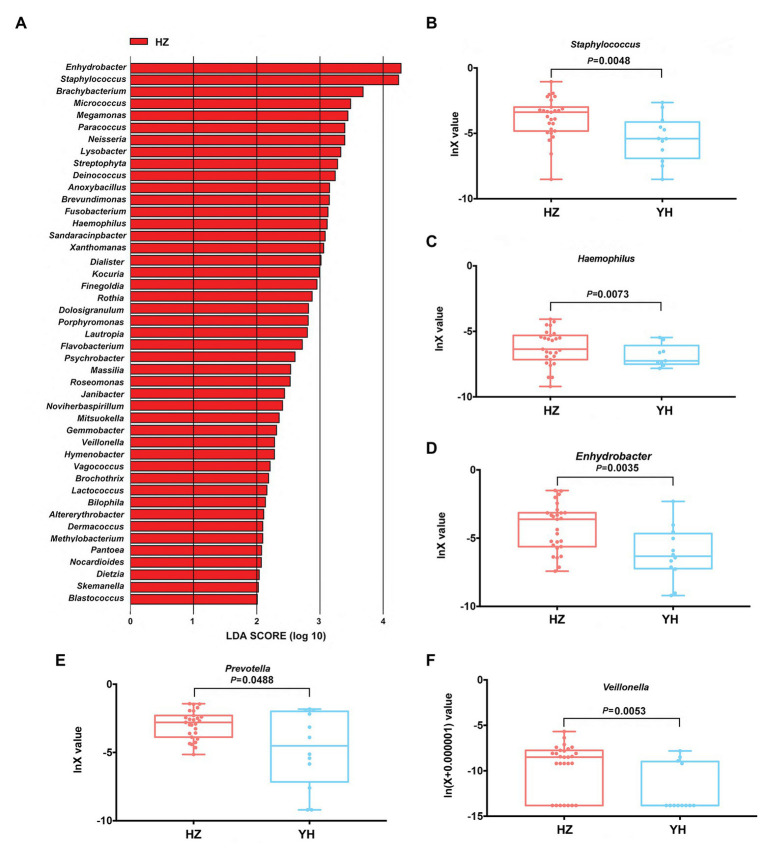
Differences in taxonomic composition of healthy women’s face between the HZ and YH groups using Lefse analysis and linear discriminant analysis (LDA). **(A)** Overexpressed bacterial genera in the HZ group chosen by LDA score > 2 (*p* < 0.05). **(B–F)** The relative expression of *Staphylococcus*, *Haemophilus*, *Enhydrobacter*, *Prevotella*, and *Veillonella* in the HZ and YH groups (*p* < 0.05). Significance determined in Wilcoxon rank sum test with one-sided alternatives.

Linear discriminant analysis (LDA) effect size (Lefse) method was further used to clarify the species that contributed to the destination-related difference in the facial bacterial microbiome between the HZ and YH groups. A total of 45 genera were chosen by LDA score > 2, all of which were overrepresented in the HZ group (*p* < 0.05, Figure 3A). Among them, *Enhydrobacter*, *Staphylococcus*, *Bachybacterum*, *Micrococcus*, *Paracoccus*, *Neisseria*, *Lysobacter*, *Streptophyta*, *Deinococcus*, and *Anoxybacillus* were the top 10 increased genera. In addition, in *Proteobacteria* phylum, *Enhydrobacter*, *Paracoccus*, *Neisseria*, *Lysobacter*, *Brevundimonas*, *Haemophilus*, *Sandaracinobacter*, and *Xanthomonas* genera were greatly enriched in HZ subjects, accounting for eight from the top 20.

Based on the previous report (Mukherjee et al., 2016), *Haemophilus*, *Enhydrobacter*, *Granulicatella*, *Streptococcus, Veillonella*, *Bacillus*, and *Prevotella* might be a hint of skin aging; we thus focused on the differential expressions of these genera between the HZ and YH groups in addition to *Staphylococcus*, one of the most predominant genera. It was shown that the expressions of *Staphylococcus*, *Haemophilus*, *Enhydrobacter*, *Prevotella*, and *Veillonella* were much higher in the HZ group than those in the YH group (*p* < 0.05, Figures 3B–F).

### Functional Changes Associated With Microbiome Changes Between HZ and YH Groups

**Figure 4:**
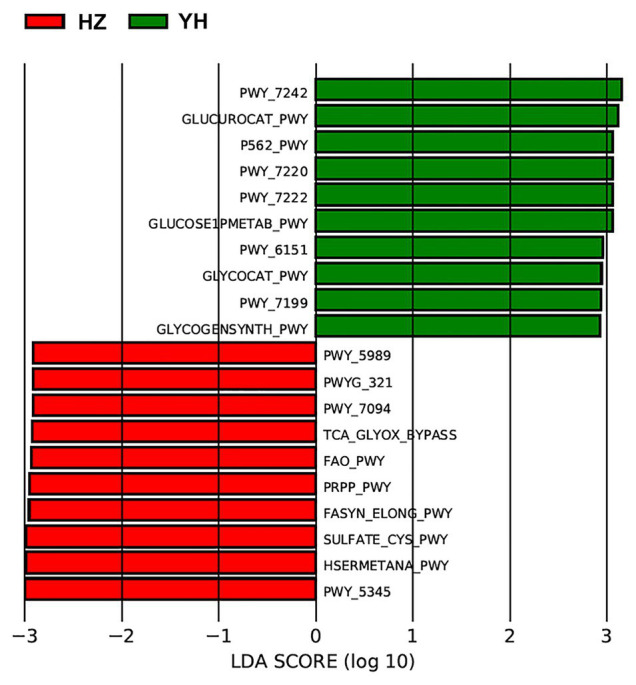
Phylogenetic Investigation of Communities by Reconstruction of Unobserved States (PICRUSt) revealed different profiles of gene function in HZ female individuals compared with YH female individuals. The red bars indicated the Kyoto Encyclopedia of Genes and Genomes (KEGG) modules dominated in the HZ group, and the green ones indicated those in the YH group (*p* < 0.05, LDA score > 2).

Using the Phylogenetic Investigation of Communities by Reconstruction of Unobserved States (PICRUSt) built-in reference database, a total of 31 Kyoto Encyclopedia of Genes and Genomes (KEGG) orthodoxies were significantly different between HZ and YH female individuals (*p* < 0.05, LDA score > 2; showing the top 10 in Figure 4). Notably, three pathways from the top 10 in the HZ group involved saturated fatty acid elongation, fatty acid β-oxidation I (generic), and fatty acid salvage, while pathways in the YH group were related to adenosine deoxyribonucleotides *de novo* biosynthesis II, guanosine deoxyribonucleotides *de novo* biosynthesis II, and pyrimidine deoxyribonucleosides salvage.

### Correlation of Facial Bacterial Microbiome Abundance With Clinical Characteristics

**Figure 5:**
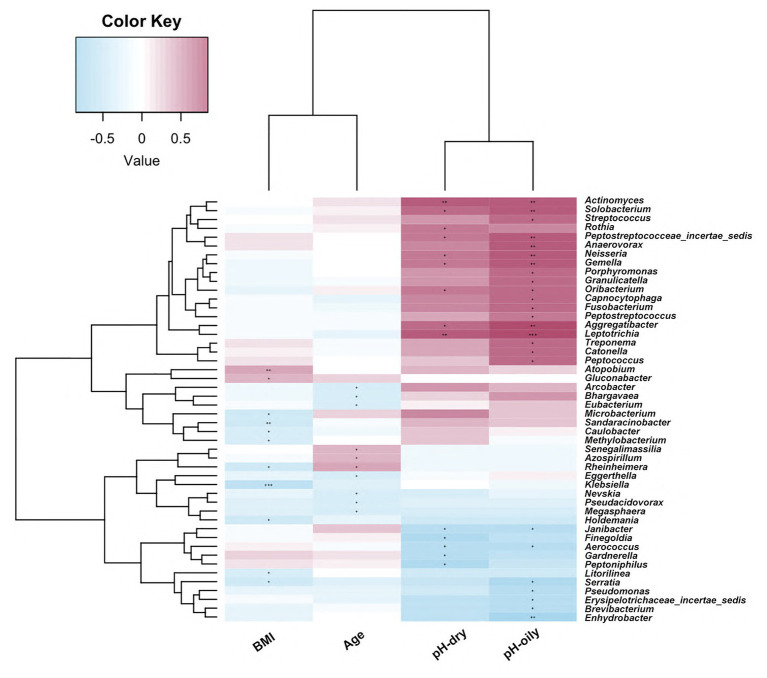
Correlations of facial bacterial microbiome abundance with clinical characteristics. The heat map of Spearman’s rank correlation coefficients between the facial bacterial microbiome abundance and clinical parameters. ^+^*p* < 0.05; ^++^*p* < 0.01; ^+++^*p* < 0.001; BMI, body mass index.

In order to identify the impact of clinical parameters on microbiome abundance, we also evaluated the correlations of the facial bacterial microbiome with clinical characteristics. As shown in Figure 5, we found that BMI was negatively correlated with *Methylobacterium*, *Serratia*, *Holdemania*, *Microbacterium*, *Sandaracinobacter*, *Caulobacter*, *Klebsiella*, and *Rheinheimera* amounts but positively correlated with *Gluconobacter* and *Atopobium* abundance (*p* < 0.05). Furthermore, age was negatively correlated with the levels of *Arcobacter*, *Bhargavaea*, *Eubacterium*, *Eggerthella*, *Nevskia*, *Pseudacidovorax*, and *Megasphaera* but positively correlated with *Senegalimassilia*, *Azospirillum*, and *Rheinherimera* (*p* < 0.05). The pH value in both oily and dry zones was positively correlated with *Leptotrichia*, *Aggregatibacter*, *Gemella*, *Neisseria*, *Oribacterium*, *Actinomyces*, and *Peptostreptococcaceae_incertae _sedis* abundance, while it was negatively correlated with *Aerococcus* and *Janibacter* abundance (*p* < 0.05).

## Discussion

In recent years, it had been reported that the diversity and abundance of skin microbiota vary with intrinsic factors, including gender, age, anatomical site, and ethnicity (Costello et al., 2009; Findley et al., 2013). External factors, such as cosmetics, soaps, and hygienic products (Lopes et al., 2015; Martin et al., 2015; Gonzalez et al., 2016; Shi et al., 2016; Urban et al., 2016), also contribute to the variation in skin microbiome. With the rapid development of national economy, environmental pollution is becoming a pressing issue. Skin, as the first line to interface with the air, is the target of various environmental stressors (Valacchi et al., 2012; Dong et al., 2019). Basic and clinical studies have provided growing evidence that airborne pollutants exacerbate skin aging (Vierktter et al., 2010; Ding et al., 2017) and even some chronic inflammatory skin diseases (Ahn, 2014; Pesce et al., 2015; Liu et al., 2018), which also exists when there is microbiome imbalance (He et al., 2006; Nielsen and Jiang, 2019). However, whether airborne pollutants have an impact on microbial communities of healthy skin, especially facial microbiome, still needs to be further explored.

Independent researches (Costello et al., 2009; Grice et al., 2009) from different regions characterized four main phyla in American population: *Actinobacteria* (36.6–51.8%), *Firmicutes* (24.4–34.3%), *Proteobacteria* (11.9–16.5%), and *Bacteroides* (6.3–9.5%). While for Chinese population, it was revealed by xi’an data (Kim et al., 2019) that *Bacteroidetes* and *Firmicutes* phyla were significantly higher in 20–30-year-old women’s face, while *Proteobacteria* and *Actinobacteria* were the top two abundant phyla in the 50–60s group. In addition, they also found that the alpha diversity of the younger group was higher than that of the older group in both species richness and phylogenetic diversity, which is in contrast to the results of a cohort of Japanese volunteers (Shibagaki et al., 2017). These discrepancies suggested that the formation of skin microbial community might be significantly affected by the individual’s residential environment and lifestyle.

Herein, focused on facial microbiome, we choose healthy and young women from two districts of Zhejiang Province in China. They have the same ethnicity, similar lifestyle, and dietary habits but with obviously different AQIs between these two districts because of different industrial development. Data of air quality collected from www.aqistudy.cn during 2014–2019 revealed a higher AQI together with higher levels of PM_2.5_, PM_10_, SO_2_, CO, and NO_2_ in HZ compared with those in YH where is there is well-developed agriculture (Supplementary Figure S3). Differed from previous report that skin’s microbial communities including bacteria, fungi, and virus were largely stable over months and years (1 month–2 years; Mukherjee et al., 2016; Oh et al., 2016), our microbiome data revealed an obvious difference in facial bacterial microbiome structure for healthy female individuals between these two districts, indicating that it might be affected by long-term exposure (>10 years) to airborne pollutes.

We further found an increase in both the taxonomic composition and the diversity of healthy female individuals in HZ. We hypothesized that similar to being inhaled into the human bodies to trigger immune response (Vawda et al., 2014) or respiratory tract infection (Miller and Peden, 2014), bacterial microorganisms, virus particles, and fungal spores (mostly sizing at the range of 0.2–2 μm) easily attached on PM particles and transferred with airflow, adhered to skin surfaces, and further caused microbiome changes. A latest study (Qin et al., 2019) reported that after exposure to high levels of PM_2.5_ and PM_10_, the abundance of the genera of *Streptococcus*, *Haemophilus*, *Moraxella*, and *Staphylococcus* in the human pharyngeal microbiome increased significantly, which is consistent with our results.

As one of the most predominant genera, *Staphylococcus* was obviously abundant in the HZ group than that in the YH group. On the one hand, *Staphylococcus epidermidis*, as a skin commensal, can activate Toll-like receptor 2 (TLR2; Lai et al., 2010) or produce phenol-soluble modulins (Cogen et al., 2010) to increase antimicrobial defense and butyric acid to downregulate the production of proinflammatory cytokines (Keshari et al., 2019). On the other hand, *Staphylococcus aureus*, usually as a potential pathogen, was found to be overgrown in atopic dermatitis (AD; Chng et al., 2016; Geoghegan et al., 2018) and could bind to TLR2 heterodimers to initiate long-lasting cutaneous inflammation (Biedermann et al., 2015). AD patients with greater *S. aureus* predominance showed more severe symptoms, while the ones with greater *S. epidermidis* predominance showed less severity (Bydr et al., 2017). Furthermore, the link between AD or eczema and pollution has been discussed before. A cohort in Southwest China (Li et al., 2018) revealed a positive association between the visits for eczema outpatients and airborne pollutants of NO_2_, SO_2_, and PM_10_, and another study from the Seoul Metropolitan Area (Kim et al., 2017) reported that a 10-U increase in PM_10_, NO_2_, and O_3_ could aggravate same-day symptoms of AD. Alpine climate treatment led to a reduction in the *Staphylococcus* genus, further resulting in significant changes in the skin microbiome composition in AD children (van Mierlo et al., 2019). However, which strain of *Staphylococcus* was the dominant one in our present study and how much of the ratio between *S. aureus* and *S. epidermidis* could indicate the risk of inflammatory skin diseases, especially atopic eczema, need to be further investigated.

As another important commensal organism of the skin, *Propionibacterium* was found abundant in both healthy individuals and acne patients (Fitz-Gibbon et al., 2013; Barnard et al., 2016). Higher relative abundances and less diversities of *Propionibacteria* and *Propionibacterium acnes* phage were found in healthy skin (Barnard et al., 2016). One study from India (Mukherjee et al., 2016) was focused on the cheek and forehead areas of young and healthy female individuals and revealed that *Propionibacterium* was positively, while *Haemophilus*, *Enhydrobacter*, *Granulicatella*, *Streptococcus*, *Veillonella*, *Bacillus*, and *Prevotella* were negatively associated with the cheek sebum that is significantly related to skin aging, which is characterized by reduced sweat and sebum secretion (Bolognia, 1995; Farage et al., 2008). Another study from Japan (Shibagaki et al., 2017) found that sebum levels were positively correlated with the *Propionibacterium* abundance, and the relative abundance of *Propionibacterium* decreased significantly in the elderly group. Both studies from Mexico (Lefebvre et al., 2015) and Shanghai (Lefebvre et al., 2016) reported that exposure to pollution could increase the facial skin dryness of individuals. More interestingly, *Propionibacterium* usually benefits the host by secreting short-chain fatty acid, antimicrobial substances, and its immunomodulation (Christensen and Bruggemann, 2014), and growth of pathogens such as *S. aureus* and *Streptococcus pyogenes* could be inhibited due to free fatty acids released by *P. acnes* (Grice and Segre, 2011). A decrease in *Staphylococcus* abundance and an increase in *Propionibacterium* abundance after coal tar treatment for AD suggested that the microbial communities of AD shifted toward those of healthy controls (Smits et al., 2020). In our study, an obvious decrease in *Propionibacterium* abundance, albeit not significant due to the small size of YH samples, together with a significant increase in *Haemophilus*, *Enhydrobacter*, *Veillonella*, and *Prevotella* was observed in the HZ group, although subjects in these two groups were age matched. Therefore, it may suggest that, to a certain extent, long-term exposure to airborne pollution would affect the physiological state of skin further to change the skin’s microbiome expression independent of chronological age.

We further identified functional variation based on the differential microbial communities between the HZ and YH group and predicted KEGG pathway on the basis of MetaCyc metabolic pathway data. The most prevalent processes of fasyn-elong-pwy, fao-pwy, and pwy-7094 in the HZ group are associated with metabolism of saturated fatty acid. Unlike unsaturated fatty acids as protective factors, saturated fatty acids exacerbate skin inflammation of keratinocytes (Herbert et al., 2018), affect the innate immune response of endothelial cells (Zhang et al., 2017), and induce cellular apoptosis (Lee et al., 2017). While in the YH group, the most dominated pathways involved the pathways for deoxyribonucleotide metabolism, such as pwy-7220, pwy-7222, and pwy-7199, which are very important for DNA repair or mitochondrial DNA replication in many types of organisms and cells (Kamiya, 2003; Setzer et al., 2008). These functional differences might provide meaningful information on the microbial role in the relationship between airborne pollution and skin microbiome communities. However, they needed to be confirmed experimentally in the future.

We also investigated the association between relevant clinical parameters with facial bacterial microbiome. As one important parameter, BMI was related with alternation in oral and vulva microbiome (Vongsa et al., 2019; de Andrade et al., 2020). A positive correlation between oral bacterial community with increased BMI was found by fingerprint analysis (de Andrade et al., 2020). High BMI (>30 kg/m^2^) was associated with higher pH in vulva and further modulated vulva microbiota, characterized by predomination of *Finegoldia* and *Corynebacterium*. However, the BMI-associated microbiota shift was not observed in abdominal skin samples (Vongsa et al., 2019). In our results, BMI was found to be negatively correlated with the abundance of several genera and positively with other two genera amounts, as shown in Figure 5. However, none of these genera differed between HZ and YH groups. It was indicated that although there was a statistical difference in BMI between the two groups, changes in facial microbiome were due to airborne pollutants rather than BMI discrepancy. In addition, similar results were also observed in the association between age and facial microbiome. A previous study (Kim et al., 2019) revealed a pattern similarity between the age type in young group of 20–30 years old and the group of 50–60 years old in the metabolism category, suggesting that an individual skin microbiome rather than chronological age might be a major determinant for skin age. As we discussed above, a decrease in *Propionibacterium* together with increases in *Haemophilus*, *Enhydrobacter*, *Veillonella*, and *Prevotella* might be a hint of skin aging. Then, in view of pH, the abundance of *Janibacter* was found to be negatively correlated with pH value and was significantly greater in the HZ group (Supplementary Table S4). However, there was no obvious difference in skin pH value in both oily and dry zones between the HZ and YH groups, which might be due to mild or moderate air pollution in HZ that is not sufficient to cause change in facial pH value or the mixed influence of free fatty acids released by *Propionibacterium* (Fluhr et al., 2001). Whether or not the increase in *Janibacter* was caused by the reduction in facial pH value as a consequence of air pollutants needs to be further verified by larger size samples.

There are some limitations to this study. The number of enrolled subjects in each group was relatively small. A total of respective 20 subjects were enrolled for each group in the beginning, while only seven oily-zone samples and five dry-zone samples from YH were used for further analysis because of the low bacterial DNA quantity obtained by swab-sampling method. Second, some skin physical parameters, such as sebum content, moisture content, and transepidermal water loss (TWEL), were not measured. The association of skin physical state and facial microbiome was not analyzed as well. Moreover, we did not perform a prospective cohort research, and whether or not air factors would have a dynamic effect on facial microbiome still remains unclear. Moreover, we noticed a higher variance of bacterial diversity and richness in YH samples. It may partly due to a little higher variance in age and BMI of YH participants and also because of a very low Shannon diversity of some samples (YH1-3, YH1-6, and YH2-10) in the YH group. An increase in sample size in our future study may reduce the impact of variance in results and conclusion.

In conclusion, this study indicated that long-term exposure to airborne pollutants could lead to significant alterations with an increase in both the abundance and diversity of facial bacterial microbiome, including metabolic pathways. Our data can provide useful information for further researches on the composition and function of the skin microbiome related to air quality as well as for the development of therapeutic agents targeting the microbes and their metabolites to resist damages of airborne pollutants.

## Data Availability Statement

The datasets presented in this study can be found in online repositories. The names of the repository/repositories and accession number(s) can be found in the article/Supplementary Material.

## Ethics Statement

The studies involving human participants were reviewed and approved by the Institutional Review Board of the First Affiliated Hospital, School of Medicine of Zhejiang University. The patients/participants provided their written informed consent to participate in this study.

## Author Contributions

XL conceived the study. YW, YZ, and XL performed the literature search and wrote the protocols. ZW, YW, YZ, and LR recruited the subjects and collected the specimens. AL performed the laboratory analyses and analyzed the data. XL and AL interpreted the data. All authors contributed to the writing and editing of the report and approved the final version.

### Conflict of Interest

The authors declare that the research was conducted in the absence of any commercial or financial relationships that could be construed as a potential conflict of interest.
