# Supplementary material for: Microbiome in Healthy Women Between Two Districts With Different Air Quality Index
Source: Front Microbiol. 2020 Oct 19;11:548618. doi: 10.3389/fmicb.2020.548618 (PMC7604314; doi:10.3389/fmicb.2020.548618)
Supplement: Supplementary file 1 [file Data_Sheet_1.pdf]

SUPPLEMENTARY DATA

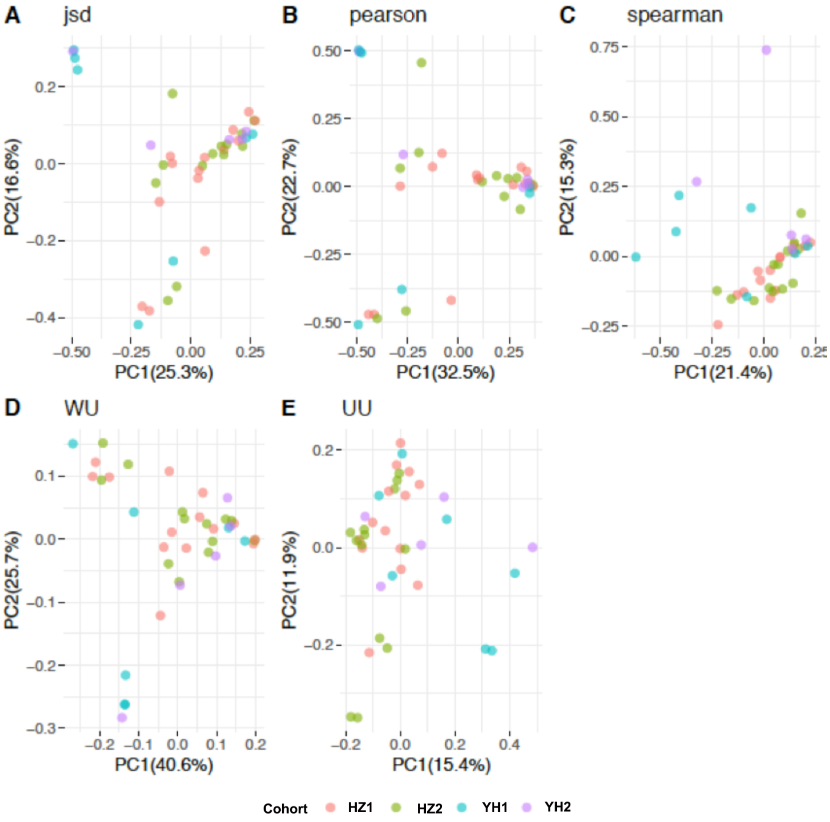

FIGURE S1 I Principle coordinates of microbial communities by principal coordinate analysis (PCoA) amongst HZ1, HZ2, YH1 and YH2 four cohorts. **(A-E)** PCoA of jsd, pearson, spearman distances, unweighted UniFrac and weighted UniFrac at the OTU level showing no significant differences in these principal coordinates between HZ and YH subjects. *HZ1 and H1 indicated data of oily-zone in the HZ district, HZ2 and H2 indicated data of dry-zone in the HZ district; YH1 and Y1 indicated data of oily-zone in the YH district, YH2 and H2 indicated data of dry-zone in the YH district.

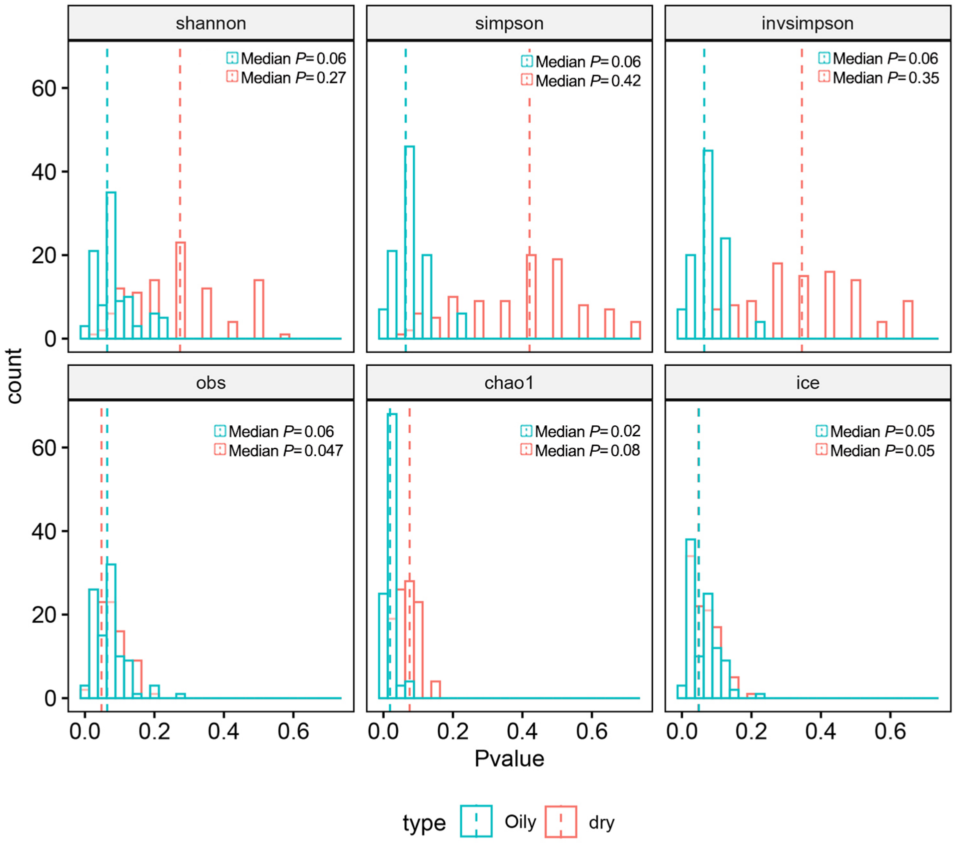

FIGURE S2 I *P* value distribution for phylogenetic diversity and richness of facial bacterial microbiome in HZ females compared to YH females using a bootstrap sampling. We randomly chose and compared 5 dry and 7 oily samples in HZ group to those in YH group, and repeated this process for 100 times. The majority of *P* value distribution is still significant. Each median *P* value was shown in the upper right corner. * Oily represented data for oily-zone, and dry represented data for dry-zone.

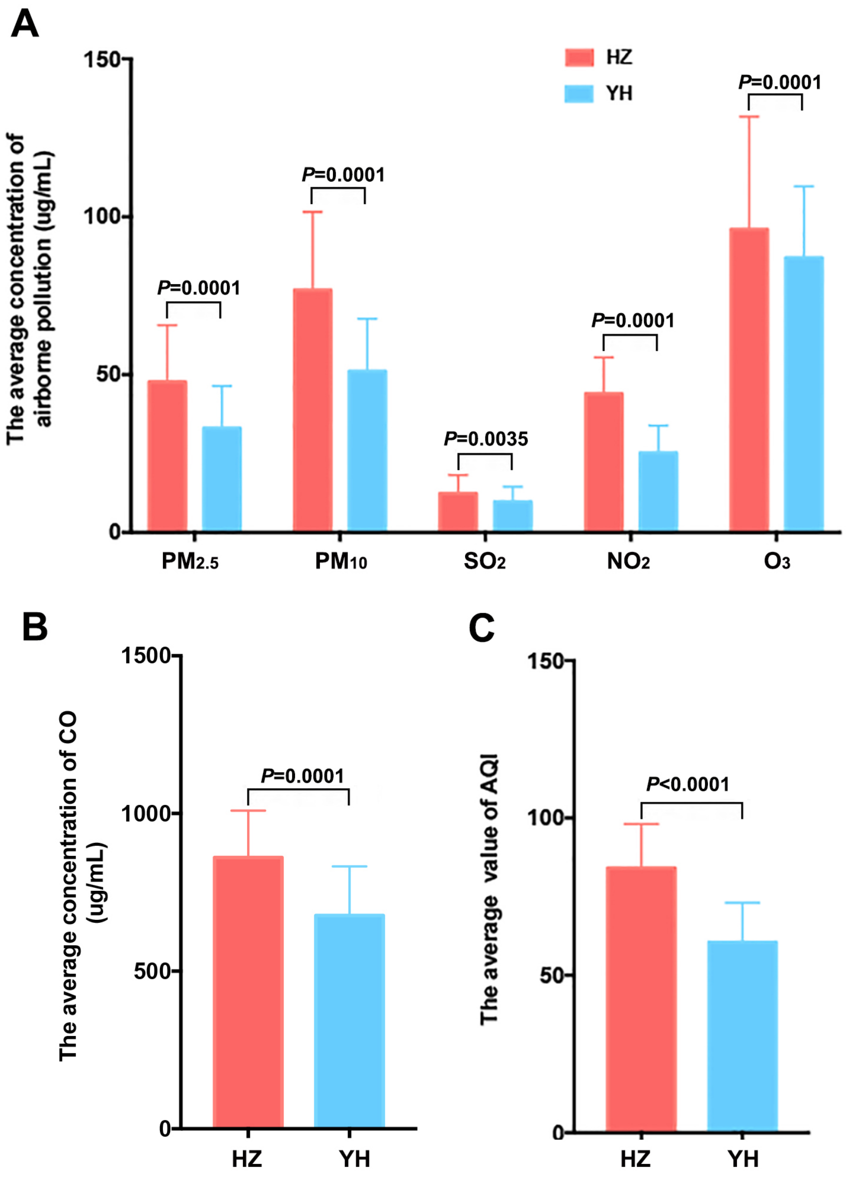

FIGURE S3 | The average values of air quality indexes in both HZ and YH ditricts during 2014-2019. **(A)** The average concentration of airborne pollution (μg/mL), including $PM_{2.5}$, PM_{10}, SO_2, NO_2 and O_3. **(B)** The average concentration of CO (μg/mL). **(C)** The average values of AQI.

**Table S1** l Exclusion Criteria (Subjects will be excluded if they have any “yes”)	
1) use of oral antibiotic or steroid within the previous 6 months;	Yes◯ No◯
2) recurrent diarrhea (>3 times), or someone with chronic diarrhea in their family；	Yes◯ No◯
3) often contact machine oil, dust, etc. in their job;	Yes◯ No◯
4) used topical antibiotic or topical steroid on the face, scalp, neck, arms, forearms or hands in the previous 7 days.	Yes◯ No◯
5) acne at sites other than on the face, chest, back or shoulders;	Yes◯ No◯
6) multiple blisters, pustules, boils, abscesses, erosions or ulcers on the scalp, face, neck, arms, forearms or hands;	Yes◯ No◯
7) a single blister, pustule, boil, abscess, erosion, ulcer, scab, cut, crack or pink/hyperpigmented patch or plaque at or within 4 cm of the sampling sites;	Yes◯ No◯
8) more than one pink/red scaly patch/plaque anywhere on the body (suggestive of psoriasis or eczema);	Yes◯ No◯
9) uniformly thickened, cracking, “dry” skin on bilateral palms and/or soles;	Yes◯ No◯
10) scalp dandruff that does not clear up with over-the-counter dandruff shampoos used daily for 2 weeks;	Yes◯ No◯
11) disseminated rash (at multiple body sites or extending throughout a broad body area).	Yes◯ No◯

**Table S2** I PERMANOVA results by using distances and characteristics of recruited individuals					
	JSD	Pearson	Spearman	Weighted Unifrac	Unweighted Unifrac
Destination (*P*)	0.002999	0.03248	0.0009995	0.02349	0.0004998
Skin-type (*P*)	0.534733	0.66267	0.3063468	0.65267	0.01999
Age (*P*)	0.538731	0.51824	0.6076962	0.85107	0.7126437
BMI (*P*)	0.374313	0.32884	0.3153423	0.46977	0.3928036
Carreer (*P*)	0.424288	0.51074	0.3348326	0.54923	0.3253373

* PERMANOVA, permutational multivariate analysis of variance.

P, referred to *P* value. Each number in each space represented a *P* value.

**Table S3** I 16S rRNA Sequencing Data from the Recrutied Subjects								
Sample	whole Num	random Num	Shannon	Simpson	Invsimpson	Obs	Chao1	Ice
H1_1	42887	10000	3.696	0.919	12.350	397	506.630	447.009
H1_2	38397	10000	4.047	0.952	21.107	415	541.068	468.708
H2_1	38634	10000	2.340	0.666	2.992	268	390.722	307.413
H2_2	41472	10000	1.594	0.444	1.799	249	375.298	299.280
H4_1	43938	10000	2.369	0.764	4.240	271	431.514	319.583
H4_2	34738	10000	4.119	0.956	22.618	363	484.440	403.873
H6_1	37640	10000	3.325	0.896	9.621	234	311.786	259.336
H6_2	35074	10000	3.831	0.913	11.503	396	497.731	444.194
H7_1	31541	10000	4.315	0.9655	28.825	383	479.040	417.679
H7_2	24238	10000	3.466	0.910	11.085	355	423.371	390.754
H8_1	38151	10000	3.944	0.940	16.585	406	579.422	460.587
H8_2	35763	10000	4.022	0.946	18.574	381	486.300	430.081
H9_2	39014	10000	3.937	0.941	16.896	424	588.377	478.202
H10_1	33307	10000	3.996	0.945	18.131	359	491.613	399.187
H10_2	24829	10000	4.701	0.973	36.732	522	654.007	568.862
H11_2	33298	10000	3.233	0.873	7.882	264	355.779	295.236
H12_1	40774	10000	3.094	0.873	7.865	225	334.796	258.581
H12_2	30251	10000	4.251	0.951	20.250	409	493.500	446.008
H13_1	19588	10000	2.094	0.693	3.263	186	269.769	212.845
H14_1	7057	7057	3.080	0.865	7.421	231	289.141	253.944
H15_1	13909	10000	3.053	0.880	8.322	218	304.327	246.447
H15_2	9471	9471	3.137	0.843	6.361	298	375.44	333.876
H16_1	6838	6838	3.698	0.908	10.888	295	431.742	336.476
H17_1	12054	10000	2.505	0.803	5.068	193	248.846	221.365
H18_2	8047	8047	3.182	0.901	10.095	243	317.014	272.703
H19_1	9073	9073	3.605	0.932	14.773	292	387.351	325.568
H20_2	11944	10000	2.381	0.726	3.647	242	338.970	275.214
Y2_1	22188	10000	1.253	0.413	1.703	143	183.500	161.632
Y3_1	50019	10000	1.091	0.433	1.762	96	129.333	119.056
Y4_1	7508	7508	3.693	0.906	10.585	291	373.047	328.354
Y6_1	48809	10000	0.590	0.162	1.193	115	147.327	133.222
Y7_2	27099	10000	2.938	0.835	6.0561	215	255.907	230.701
Y9_2	10738	10000	3.358	0.921	12.718	214	274.017	237.228
Y10_2	44328	10000	0.350	0.128	1.147	57	144.500	92.694
Y13_1	10916	10000	3.353	0.897	9.726	235	304.231	256.339
Y16_2	9789	9789	3.774	0.929	14.008	295	339.947	317.295

Y18_1	47619	10000	0.715	0.250	1.333	114	177.84	143.011
Y18_2	9089	9089	4.094	0.954	21.851	371	520.398	421.493
Y20_1	11083	10000	3.235	0.897	9.695	314	379.921	343.574

* In the list of sample, H indicated samples from HZ and Y indicated samples for YH. The first number of “1, 2, …, 20” referred to the serial number of subjects, and the second number of “1” or “2” referred to sample from the oily-zone or the dry-zone.

Table S4 I Genera to construct random forest models to distinguish HZ and YH females

Genus	Mean (HZ)	Mean (YH)	*P* value	Enrich
Paracoccus	0.005137793	6.16E-05	1.83E-06	HZ
Psychrobacter	0.000906475	2.75E-05	0.000219059	HZ
Massilia	0.000705038	8.83E-06	0.000535797	HZ
Hymenobacter	0.000387527	0	0.000559252	HZ
Micrococcus	0.007550544	0.000142478	0.000685417	HZ
Skermanella	0.000160807	0	0.000937829	HZ
Altererythrobacter	0.000261359	8.42E-06	0.001221017	HZ
Lysobacter	0.004596937	2.61E-05	0.001243248	HZ
Sandaracinobacter	0.00252164	1.87E-05	0.00215203	HZ
Lautropia	0.001584694	0.000193236	0.002432929	HZ
Blastococcus	0.000177572	0	0.002471432	HZ
Noviherbaspirillum	0.00051986	0	0.002471432	HZ
Janibacter	0.000700575	0.000136295	0.002742194	HZ
Brachybacterium	0.00934188	6.20E-05	0.00277972	HZ
Streptophyta	0.003822833	0.000317166	0.003369501	HZ
Enhydrobacter	0.045245956	0.01196326	0.003584353	HZ
Brochothrix	0.000299988	0	0.003893731	HZ
Roseomonas	0.000697438	0.000109281	0.004038702	HZ
Kocuria	0.002150396	0.000210097	0.004148522	HZ
Neisseria	0.005922807	0.001142591	0.004184784	HZ
Staphylococcus	0.051497569	0.014559535	0.004819044	HZ
Pantoea	0.000196771	1.68E-05	0.004851061	HZ
Xanthomonas	0.002266464	3.41E-05	0.005132681	HZ
Veillonella	0.000398192	6.97E-05	0.00533408	HZ
Brevundimonas	0.003393447	0.000390691	0.005753899	HZ
Dietzia	0.000193898	0	0.006023762	HZ
Rhizobium	6.70E-05	0	0.006023762	HZ
Lactococcus	0.000284662	3.77E-05	0.007215233	HZ
Deinococcus	0.004050538	0.000361703	0.007315724	HZ
Haemophilus	0.003912761	0.001123282	0.007768841	HZ
Flavobacterium	0.001186996	0.000135118	0.008129348	HZ
Bilophila	0.000307422	0.000152143	0.011154524	HZ
Nocardioides	0.000242298	1.77E-05	0.012754839	HZ
Gemmobacter	0.000406413	8.41E-06	0.013472296	HZ
Arsenicicoccus	0.000126083	0	0.013723676	HZ
Anoxybacillus	0.00509425	0.002423956	0.014221844	HZ
Mitsuokella	0.00072431	0.000359852	0.014559641	HZ
Dialister	0.004534873	0.002988468	0.014773284	HZ
Rothia	0.003039239	0.001634734	0.014781521	HZ
Methylobacterium	0.000443606	0.000238811	0.015790035	HZ

Dolosigranulum	0.001556693	0.000192767	0.015790035	HZ
Finegoldia	0.002477999	0.000688773	0.015814957	HZ
Dermacoccus	0.000251868	6.82E-05	0.015999512	HZ
Anaeroplasma	0	4.87E-05	0.017461334	YH
Moraxella	0.001273578	8.37E-05	0.019483739	HZ
Cellulomonas	6.47E-05	0	0.020268981	HZ
Megamonas	0.01039581	0.005499086	0.023014793	HZ
Porphyromonas	0.00206456	0.00092326	0.02524496	HZ
Vagococcus	0.00045082	1.12E-05	0.025351192	HZ
Fusobacterium	0.003874088	0.001337947	0.025680913	HZ
Sutterella	0.000796111	0.000488721	0.026327302	HZ
Butyricicoccus	0.000162247	8.92E-05	0.027274699	HZ
Acinetobacter	0.018262535	0.008026	0.028207128	HZ
Novosphingobium	0.00108231	0.001455668	0.028549415	HZ
Marmoricola	0.000246709	2.05E-05	0.029156368	HZ
Streptobacillus	0.000679718	0	0.029569869	HZ
Mobilicoccus	5.63E-05	0	0.029569869	HZ
Microvirga	3.90E-05	0	0.029569869	HZ
Sphingobacterium	9.56E-05	0	0.029569869	HZ
Dyadobacter	5.66E-05	0	0.029569869	HZ
Corynebacterium	0.01656285	0.014825699	0.032547377	HZ
Flavonifractor	0.000521798	0.000358263	0.03393736	HZ
Anaerococcus	0.002205739	0.000988066	0.034256056	HZ
Abiotrophia	0.001211463	0.000380682	0.035425124	HZ
Rubellimicrobium	0.000480132	4.43E-05	0.036004405	HZ
Aggregatibacter	0.000712878	0.000231519	0.036280965	HZ
Sphingomonas	0.003596895	0.001529175	0.036287469	HZ
Clostridium_XlVb	0.000738824	0.000395182	0.039170633	HZ
Bradyrhizobium	0.00039895	0.000221725	0.039902956	HZ
Bacteroides	0.045905829	0.029835586	0.040041039	HZ
Anaerotruncus	5.67E-05	1.87E-05	0.041754697	HZ
Parasutterella	0.001133119	0.000810798	0.042341882	HZ
Parasegetibacter	4.54E-05	0	0.042695697	HZ
Coprobacter	3.93E-05	0	0.042695697	HZ
Labilithrix	2.62E-05	0	0.042695697	HZ
Epilithonimonas	2.62E-05	0	0.042695697	HZ
Modestobacter	5.66E-05	0	0.042695697	HZ
Myroides	0.000691337	0	0.042695697	HZ
Bifidobacterium	0.023892593	0.015947147	0.042820562	HZ
Pseudomonas	0.005112069	0.002653653	0.045595039	HZ
Gemmiger	0.003354501	0.002675712	0.045749064	HZ
Prevotella	0.079730572	0.054044016	0.048831232	HZ
Propionibacterium	0.017862401	0.294321528	0.149153971*	YH

* $P > 0.05$
